# BurnCare tablet trainer to enhance burn injury care and treatment

**DOI:** 10.1186/s12873-020-00378-z

**Published:** 2020-10-30

**Authors:** Austin Baird, Maria Serio-Melvin, Matthew Hackett, Marcia Clover, Matthew McDaniel, Michael Rowland, Alicia Williams, Bradly Wilson

**Affiliations:** 1 Applied Research Associates, Inc., 8537 Six Forks Rd, Raleigh, NC 27615 USA; 2 USARMY Institute of Surgical Research, 3698 Chambers Pass Ste B JBSA ft. Sam, Houston, TX 78234-7767 USA; 3 Army Research Laboratory, 12423 Research Pkwy, Orlando, FL 32826 USA

**Keywords:** Burn, Simulation, TBSA, Escharotomy, Training, Android, Tablet, Treatment, Military

## Abstract

**Background:**

Applied Research Associates (ARA) and the United States Army Institute of Surgical Research (USAISR) have been developing a tablet-based simulation environment for burn wound assessment and burn shock resuscitation. This application aims to supplement the current gold standard in burn care education, the Advanced Burn Life Support (ABLS) curriculum.

**Results:**

Subject matter experts validate total body surface area (TBSA) identification and analysis and show that the visual fidelity of the tablet virtual patients is consistent with real life thermal injuries. We show this by noting that the error between their burn mapping and the actual patient burns was sufficiently less than that of a random sample population. Statistical analysis is used to confirm this hypothesis. In addition a full body physiology model developed for this project is detailed. Physiological results, and responses to standard care treatment, are detailed and validated. Future updates will include training modules that leverage this model.

**Conclusion:**

We have created an accurate, whole-body model of burn TBSA training experience in Unreal 4 on a mobile platform, provided for free to the medical community. We hope to provide learners with more a realistic experience and with rapid feedback as they practice patient assessment, intervention, and reassessment.

## Background

Burn injuries are a serious life-threatening injury to the warfighter in a combat zone. In Operations Iraqi and Enduring Freedom (OIF & OEF) burns comprised 5% of casualties evacuated from the battlefield [1]. These injuries result in approximately a 4% mortality rate, but the compounding effects of the recovery can lead to life-long reductions in quality of life. Evacuation time from the battlefield to increasing levels of care decreases with increasing burn size, but during OIF and OEF evacuation time to the US Army Burn Center took on average 4 days for patients with burns of over 40% of their body [2]. Compounding this evacuation time is the continuously evolving battlefield with increasing far forward disparate engagement zones. The Department of Defense (DoD) anticipates the future battlefield to consist of large-scale combat operations across multiple domains, with near peer adversaries resulting in a lack of air and ground superiority and significantly delayed evacuation to definitive care [3]. This creates a need for more burn care training focused on prolonged settings. A patient with over 40% total body surface area (TBSA) burn is and will be in critical need of constant monitoring and resuscitation for at least the first 3 days following the event. If evacuation is denied or delayed for many hours a more diverse set of care providers, inexperienced with burn care, must be able to diagnose, treat, stabilize and continue care for a critically wounded burn patient [4].

In addition to systemic challenges, there are practical challenges to caring for burn patients. Casualties with burns involving greater than 20% TBSA require careful resuscitation in the first 24 to 48 h after injury. However, the person initially treating the casualty (as well as the next several providers in the evacuation chain) will likely not be physicians or nurses who specialize in burn care and may not be familiar with proper resuscitation procedures. To mitigate the problem of inexperience, in 2006, the Burn Care clinical practice guideline (CPG) was developed by burn providers at the United States Army Institute of Surgical Research (USAISR) Burn Center and disseminated via the Joint Theater Trauma System, to provide recommendations for optimal care of patients burned while involved in combat operations. Improvement in patient outcomes has been demonstrated when providers adhere to guidelines and utilize the burn flow sheet, but repeated and specialized training is still needed [5, 6].

The unique physiological response exhibited by a patient with large surface area burns exemplifies need for specialized training at every level of patient care. Developed by the American Burn Association (ABA), the Advanced Burn Life Support (ABLS) Provider course prepares the care provider for the first 24 h post burn injury.

ABLS is comprised of didactic presentations, case scenario hands-on practice, concluding with a written exam and practical hands-on skill test and is offered periodically by specialty trained ABLS Course instructors. These courses have been shown to provide participants with improved understanding of the complex requirements of burn treatment and to be positively supplemented by high fidelity simulations [7], however, not nearly enough non-burn care clinicians, to include the military providers, are able to attend the course. For example, according to the ABA, only 245 clinicians in North Carolina had completed ABLS between 2000 and 2007 [8]. This is in contrast to the over 60,000+ nurses in critical or emergency medicine in the state. Participation in ABLS is vastly underwhelming and only demonstrates the need for more wide-spread, affordable, and easily accessible training [9].

Our intent is to provide a mobile, tablet-based burn care simulator which can augment ABLS or facilitate familiarization for any care provider involved in thermal injury treatment. As provided, BurnCare provides refresher training on determining burn size (TBSA burned). We detail development of the software and validate the fidelity of the virtual patients created for this effort. In addition, we detail a thermal injury burn physiology model currently being incorporated into the BurnCare training game. We discuss a validated treatment protocol using the BioGears computational model that includes fluid resuscitation and pain management. This model is freely available through the biogears engine source code (link) and will be incorporated into the BurnCare training platform in the near future, resulting in physiologically realistic burn care simulations and enhanced training experiences for the user. Future module updates will include burn resuscitation, escharotomy, pain management, and prolonged multi-trauma care.

## Implementation

### Unreal engine tablet burn care training application

The BurnCare training application is developed using Unreal Engine 4 because of the out of the box multi-platform support (for applications beyond Windows operating systems) and its built-in scaling features. These features were critical for development because this provided automated resolution scaling across user platforms. Touch controls and camera movement across the three dimensional domain were developed in house and rigorous play testing provided the proper acceleration and navigational prompting. The user may automatically snap to a specific bodily location using a provided side button and zoom in/out via traditional pinch controls. Additional buttons allow for translational movement across the specified virtual patient limb. Background domains and initial patient configurations were all specified by SME burn care domain experts, to mimic treatment in a prolonged care setting. This is a setting where evacuation to specialized trauma support may not be available to the patient. Generally in these settings, less trained care providers would need to be in charge of patient care for over 24 h. This setting provides motivation for the learning objectives of the tablet BurnCare application.

**Fig. 1:**
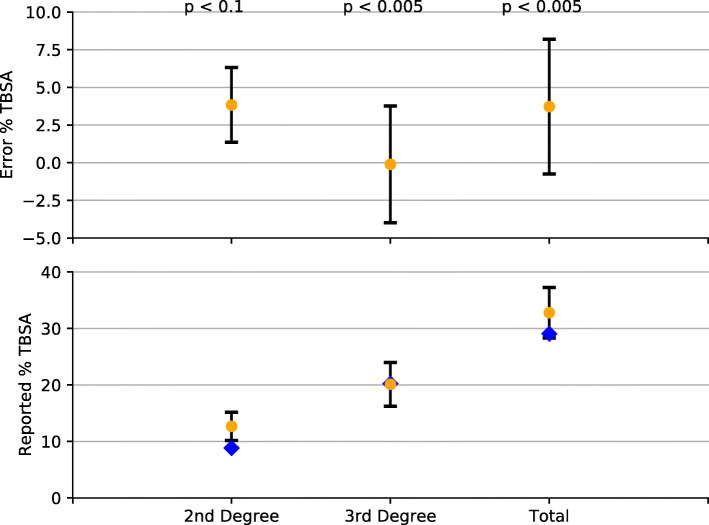
Results of TBSA estimation provided by USISR SMEs. Error is computed as the difference between the generated burn surface area in the game and the reported TBSA by the SMEs. Bars around the data indicates one standard deviation from the mean. The blue diamonds on the reported TBSA is the computer generated burn surface area. Data is reported as a mean (and standard deviation) of 10 random virtual patient trails where SMEs reported TBSA on each generated patient

The tablet game is developed in a modular fashion, allowing for each module to train the user on a specific skill relevant to burn care. The purpose of the first module is to test a clinician on a key burn skill, accurately determining the TBSA burned by correctly coloring a Lund and Browder (LB) diagram and filling out the LB table. In order to develop this module, USAISR burn center SMEs volunteered their time to use the software and provide feedback. Initial virtual burn renderings were updated and developed with hundreds of reference images and proper clinician feedback. After the game renderings were deemed accurate, a randomization algorithm was developed to spread the burn across the body of the simulated patient. Accuracy of the images were tested through a collaboration with USAISR, Fig. 1.

The BurnCare training application currently has one main module that seeks to teach how to determine the patient’s TBSA burned. The modularity of the overall application creates the ability to increase training size and scope with additional effort. This module has been identified as a critical first step and is unique to burn wound care with which providers often have limited experience. Accuracy of TBSA assessment leads to maximizing patient outcomes. This module, and all future modules, has been shaped by USAISR subject matter experts (SMEs) to incorporate relevant detail, common pitfalls, and as much realism as possible. The scenario presented to the user upon patient arrival is a military Service Member (SM) covered with dirt and blood. It is verbally communicated that the patient was in a dismounted improvised explosive device (IED) blast and has suffered severe burn wounds as a result. It is assumed that the player is in an austere role 2 facility and it has been communicated that evacuation has been denied up to 24 h. After the primary and secondary survey are complete, the next step is to identify the TBSA suffered in order to determine the amount of intravenous fluid the patient will need for burn fluid resuscitation and other specialized burn care treatments. The intent for future development is incorporating additional trauma that must be managed appropriately while administering burn fluid resuscitation. All training is developed for an android tablet and will be provided for free through the Google Play store, future releases may target IOS devices.

**Fig. 2:**
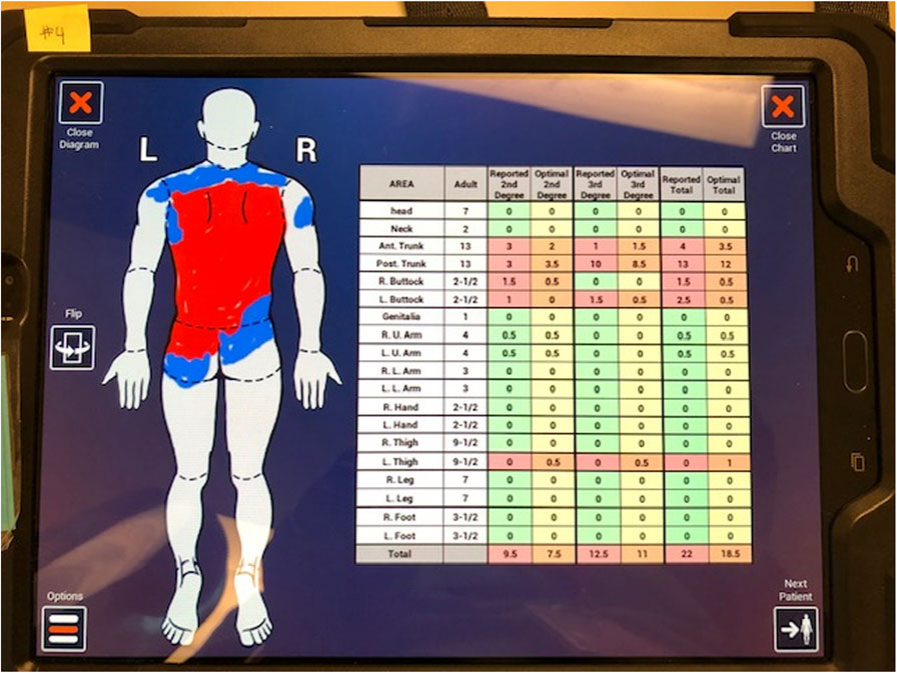
A burn care clinician finishes properly documenting the virtual Lund-Browder chart, effectively mapping the patients partial and full thickness burns. This task was completed by 9 care providers to provide data to support the visual fidelity of the models used in the game

Clinicians mapped five virtual patient’s injuries on the digital LB diagram and table in the software. They colored full thickness burns as red and partial thickness burns as blue on the corresponding body part on the LB diagram. Secondly, they determined the percent body surface area burned by estimating the percent burned of that body part and writing the number in the correct row and column of the LB table provided in the application (See Fig. 2). Afterwards, they had to add the numbers in the rows and columns to determine the percent partial and full thickness burned as well as the TBSA burned. After the clinician pressed the “Finish Estimate” button, the software provided the clinician with the computer’s answers, allowing for after action review and appropriate re-enforcement training.

#### Character generation

For realistic looking virtual patients, we used actual body scans to create the meshes for our 3D character models. We chose to implement five male and five female characters of different body-types, from skinny to obese, and apply different skins and faces to these character meshes, allowing us to generate dozens of variations per character mesh. Burn textures, encompassing partial and full-thickness burns, are procedurally generated and randomly placed on the characters, multiplying exponentially the number of virtual patients and re-playability value of the BurnCare mobile application. All ten basic character meshes contain a physically realistic bone structure to allow for anatomically accurate animations when manipulating the virtual patient.

#### Tablet controls and gameplay

**Fig. 3:**
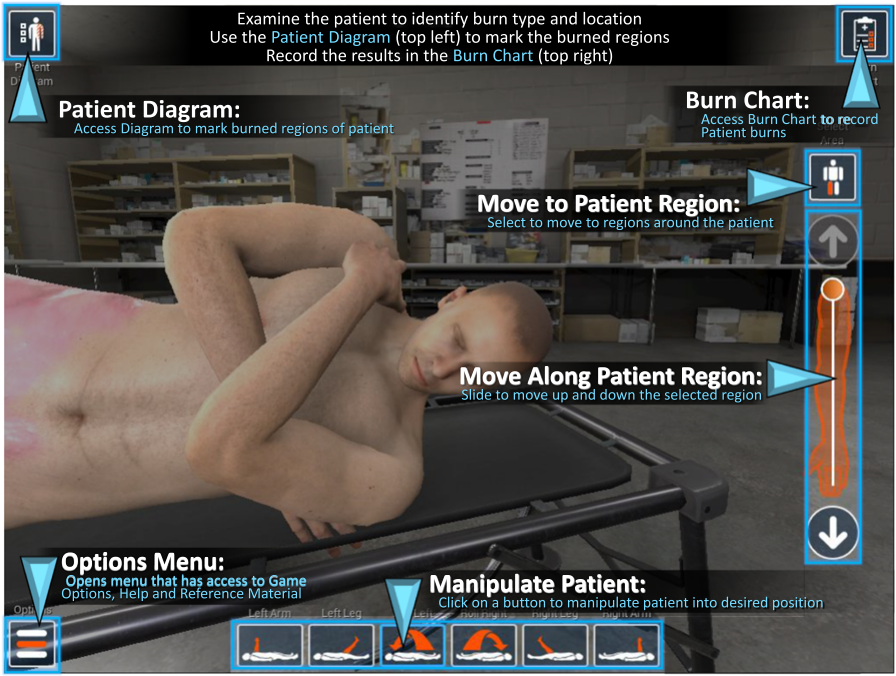
Tablet controls limit the number of buttons available to the user while playing BurnCare. To overcome this obstacle, time was spent configuring the screen, camera and interact able icons to improve the user experience

The player sees the world through the eyes of his or her character and can easily interact with the application controls by means of tapping and touch and drag movement on the tablet. The controls of the game are designed to be extremely accessible to players with all levels of computer experience to minimize barrier to entry for player learning. Interacting with on-screen menu elements consist of simple touch mechanics. In addition, each game screen displays onscreen text to aid in understanding and using the available interface elements. See Fig. 3.

When the player taps on an interactive element, they perform the specified option (e.g., reposition patient, move camera, open chart, etc.). The player is able to examine, and manipulate, the patient within a restricted amount by means of touching and dragging within the virtual environment. Within the TBSA module, the player is responsible for thoroughly examining the patient and reporting the accurate corresponding burn percentage and type. Future modules will have different player requirements unique to the burn care best practice guidelines and developed with guidance from USAISR SMEs.

### BioGears burn model

The BioGears Engine (https://www.biogearsengine.com) is a whole-body physiological model designed for accurate simulation of numerous medical insults, interventions, and conditions. Zero-dimensional electrical circuit analogs describe the cardiovascular and respiratory systems, promoting speedy computation of pressures, flows, and volumes. BioGears subdivides these systems into compartments (e.g. liver vasculature, muscle tissue, etc.) for greater specificity. Substances such as blood, proteins, ions, and drugs circulate through the model via convection and diffusion. Physics-based feedback models control the response of the system to perturbations and maintain homeostasis within physiological bounds. A BioGears engine burn physiology model increases physiological accuracy of the developed application and will be the backbone of future development on this project. This model is currently integrated into the BioGears physiology engine, provided free under the Apache 2.0 open source licensing agreement, and is being tested and integrated into the tablet application for additional module development.

Hyper-inflammation drives the pathophysiology of thermal injury [10–12], the severity of which correlates well with TBSA burned [12]. BioGears maintains a dynamic model of acute inflammation based on the work of Chow [13], Reynolds [14], and Dominguez-Huttinger [15] that accounts for interactions between numerous pro- and anti-inflammatory mediators associated with burns—such as tumor necrosis factor (TNF) and interleukins-6 and 10 (IL-6, IL-10) [10, 14]—and their collective contribution to endothelial damage. We have described the BioGears inflammation model previously in an in silico study of sepsis onset and treatment [6]. We calibrated this model to burn-mediated inflammation by setting all infection states to zero and introducing a thermal trauma term to the equations describing blood macrophage, blood neutrophil, and tissue integrity kinetics (Equations 6–9, 18 in [6]).

**Fig. 4:**
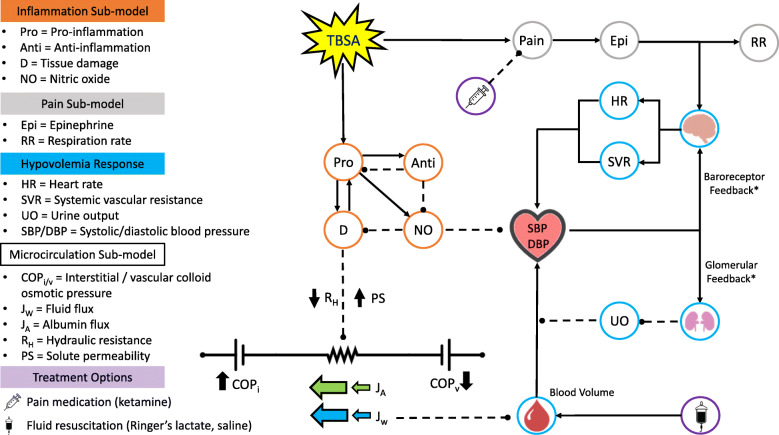
Process flow of the BioGears inflammatory model in response to thermal injury. A TBSA input initiates the inflammatory cytokine kinetic model and generates a pain signal. The pain signal upregulates epinephrine production, which increases respiration rate and acts upon the BioGears cardiovascular model. The interaction of the pro- and anti-inflammatory compounds effects the integrity of the tissue (D) and produce the vasodilator nitric oxide (NO). The effects of tissue damage are modeled by reducing hydraulic resistance and increasing solute permeability on the connecting the vascular and interstitial regions of the BioGears cardiovascular circuit. These changes favor fluid loss to the interstitium, resulting in decreased blood volume and blood pressure. The baroreceptor and glomerular feedback models work to preserve blood pressure by increasing heart rate and systemic resistance and decreasing urine output

We initiate a burn action in BioGears by specifying the size of the wound in terms of TBSA. This input upregulates the production of pro-inflammatory cytokines, which release factors that degrade the endothelial barrier and produce free radicals like nitric oxide, Fig. 4. An anti-inflammatory response opposes these actions and prevents excessive damage accumulation in the case of smaller burns. Large burns, however, cause a disproportionately large pro-inflammatory response that cannot be completely checked by anti-inflammatory mediators. We model endothelial barrier deterioration by decreasing the resistance of each pathway that links a BioGears vascular compartment to its companion extravascular compartment. Volumetric flux to the interstitium increases accordingly, accompanied by enhanced filtration of blood substances—most notably albumin, which BioGears uses to update colloid osmotic pressure (COP) on the cardiovascular circuit [16]. The deterioration of the albumin concentration gradient reduces BioGears COP, exacerbating fluid loss and leading to the massive plasma volume depletion observed in large burns. Vascular volume depletion initiates a cascade of BioGears feedback that models other aspects of burn pathophysiology.

The BioGears baroreceptor model responds to developing hypotension by increasing heart rate and systemic vascular resistance. Similarly, the BioGears renal system conserves fluid by downregulating glomerular filtration and increasing reabsorption, causing a decrease in urine output. We furthermore assume that the burn causes a massive pain response. BioGears includes a generic pain model that upregulates epinephrine production—driving further sympathetic outflow via the BioGears drug system—and increases respiration rate.

We validate the BioGears burn model by simulating untreated burns of 10, 25, and 40% TBSA. We then repeat the 25% TBSA burn scenario and intervene using protocols outlined in [17, 18]. These protocols call for initial infusion of Lactated Ringer’s solution at 10 * TBSA mL/h, titrated up or down by 25% every hour to achieve a urine output in the range 30–50 mL/h. They also recommend administering a ketamine bolus of 20 mg to manage pain. Both of these actions are supported by BioGears and all output data was reviewed by burn care surgeons at USISR.

### Statistical analysis

We sought to determine the visual fidelity of the virtual representation of the burn by leveraging health care providers at USISR. We hypothesized that an average user determining TBSA using the BurnCare application would be able to compute 2nd and 3rd degree distinctions within 5% and the total burn surface area within 10%. Effectively we test: *H*_0_ : *μ* = 10 or *H*_*A*_ : *μ* < 10 for the total burn mapping. Further refinement of the population mean may be an area of future efforts. If the visual fidelity of the application was sufficient, then health care provider experts on burn care would be able to determine virtual patient TBSA at a more efficient rate. We then seek to test this null hypothesis for each of the 2nd, 3rd, and total degree burn mapping on the Lund-Browder chart.

Rigorous statistical analysis of the BurnCare tablet training application, used in an ABLS course will be reported in future studies. For this study, we aimed to construct a sample population of health care providers who we consider to be the gold standard of TBSA assessment, having worked extensively in a burncare facility. We randomized a sample of 9 providers across health care roles: clinicians, nurses, and physician assistants. For each of these users, we have them fill in the attached Lund-Browder chart for a random virtual burn patient. We then assume a Gaussian mean distribution over these health care providers and compute a mean and standard deviation that is related to determining TBSA on the BurnCare tablet game. We test the null hypothesis by determining the t statistic for 2nd 3rd and total degree burn reported for this population then use this value to determine the *p*-value for each reported metric. Mean, standard deviation, t and *p* values are manually computed using python leveraging the Scipy statistic libraries. Code used to generate these statistics and the associated data can be found here.

## Results

### Tablet TBSA training application

The TBSA training module presents the user with a randomized patient and a randomized set of partial and full thickness burns in order to increase repeated training and diversity of assessments required by the user. This exercise tasks the user with determining the burn surface area and filling out the appropriate paperwork (paper Lund & Browder table and chart found in the Burn Care CPG) to aid in this estimation and to enhance in down-stream handoffs such as aeromedical evacuation. In order to increase the use of the application, the system has been designed to be used by all levels of clinicians on the forward resuscitation surgical team (FRST(−).) Animations and touch controls aid the users’ assessment and requires them to determine injuries on the entire body, not just the anterior surface. For example, tabs allow the user to turn the patient and lift extremities to allow for a more realistic and accurate assessment of the burns.

In the early stage of development, eight (4 surgeons, 2 physician assistants and 2 clinical nurse specialists) USAISR Burn Center SMEs used the TBSA training module and provided feedback that was used to improve the software. The SMEs used the module to determine the TBSA burned on five virtual patients. Each of the eight clinicians mapped the virtual patient’s injuries on the digital Lund and Browder (LB) diagram and table in the software, Fig. 2. They colored full thickness burns as red and partial thickness burns as blue on the corresponding body part on the LB diagram. Secondly, they determined the percent body surface area burned by writing the number in the correct row and column of the LB table exactly as would be done on the form provided in the burn CPG. Afterwards, they added the rows and columns to determine the percent partial and full thickness burned along with the TBSA burned. After the clinician pressed the “Finish Estimate” button, the software provided the clinician with and “after action report” showing them the “correct” answers, generated by the computer’s software. Estimated and actual burn surface area data is reported in Table 1. Performance between the burn SMEs and the simulated TBSA is provided in Fig. 1.

**Table 1:** Data generated by health care providers on a randomized list of 9 patients. Reported denotes the value of 2nd and 3rd degree burn TBSA, using the Lund-Browder chart, the provider mapped from the generated virtual burn patient. Generated denotes the exact surface area that the burn texture was applied to the virtual patient

Picture ID	Reported 2nd	Generated 2nd	Reported 3rd	Generated 3rd	Reported Total	Generated Total
**1**	13.5	11.5	31	33.5	44.5	45
**2**	17	8.5	25.5	25	42.5	33.5
**3**	15	11.5	37	33.5	52	45
**4**	3	3.5	5	4.5	8	8
**5**	10.5	7.5	13.5	11	24	18.5
**6**	17.5	12.5	11	13.5	28.5	26
**7**	14.5	8.5	29.5	25	44	33.5
**8**	9.5	7.5	12.5	11	22	18.5
**9**	13.5	8.5	16	25	29.5	33.5

Overall accuracy of the SMEs was quite high with total error mean averaging around 3 percentage points. Because the users were highly trained burn care providers, these results showcases the visual accuracy of the burns on the virtual patient. A normal distribution was assumed and associated standard deviation was calculated and reported, evaluation distinctions were made between estimations of third, second, and total degree burn estimations, Fig. 1. The statistics involved in the visual accuracy study are presented in Table 2 . Here the mean denotes the mean of the difference, or error, between the SMEs and the ground truth for determining TBSA as a percentage out of 100. 2nd degree burn did not meet the desired *p*-value of < 0.005. This shows that the visual fidelity of the 2nd degree burns in the application could be adjusted to be more accurate and will be an area of future improvements.

**Table 2:** Statistics reported to test the visual fidelity of the application. Strong results show that SME users are able to determine burn wounds at a much lower error rate than an average population sample. 2nd degree burns show less strong correlation to support this hypothesis

Burn	Mean (%)	Standard Deviation	t-statistic	*p*-value
2nd Degree	3.8	2.5	−1.4	0.098
3rd Degree	−0.1	3.9	−3.96	0.0021
Total	3.7	4.5	−4.2	0.0014

Overall, the SMEs were pleased with the applications performance and could imagine using it to enhance training of non-burn experienced clinicians at any level. Many stated that the software was intuitive and easy to use and that burn injuries looked realistic. The actions needed to color the Lund and Browder (LB) diagram and fill out the LB table in the software, were very similar to filling out the paper version in an actual clinical environment, requiring the same amount of thinking as when they performed this task on a real patient. Several recommendations were provided to improve the software such as improving the slider button to more effectively be able to lift extremities and change the view of the patient on the screen in order to assess their burn wounds more effectively. The most common request for the software was to automatically add the numbers in the rows and columns of the LB table, while the table was being filled out.

Post- game analysis of the SME data comparing their estimation of TBSA burned with the computer-generated number showed that SME estimation of burn was very close to actual virtual patient burn (as administered in the simulation), Fig. 1. Overall, second degree burns were estimated within 4% of actual, third degree burns were estimated almost exactly (with high variance), and total evaluation of the TBSA of the virtual patient was documented within 4%. A normal distribution was assumed and associated standard deviation was calculated and reported. Estimation of total TBSA by the SME was over reported but the actual value was within one standard deviation of the data.

This shows that the visual accuracy of the burned patient is high and the process of filling out the Lund Browder chart is fairly easy to use and mimics the process SMEs have experienced in real life. This information is being used to improve the module by providing after action report data. If a user scores within a SME mark up of a virtual patient (or within one standard deviation) that would be considered a successful TBSA assessment of the patient.

### BioGears results

**Fig. 5:**
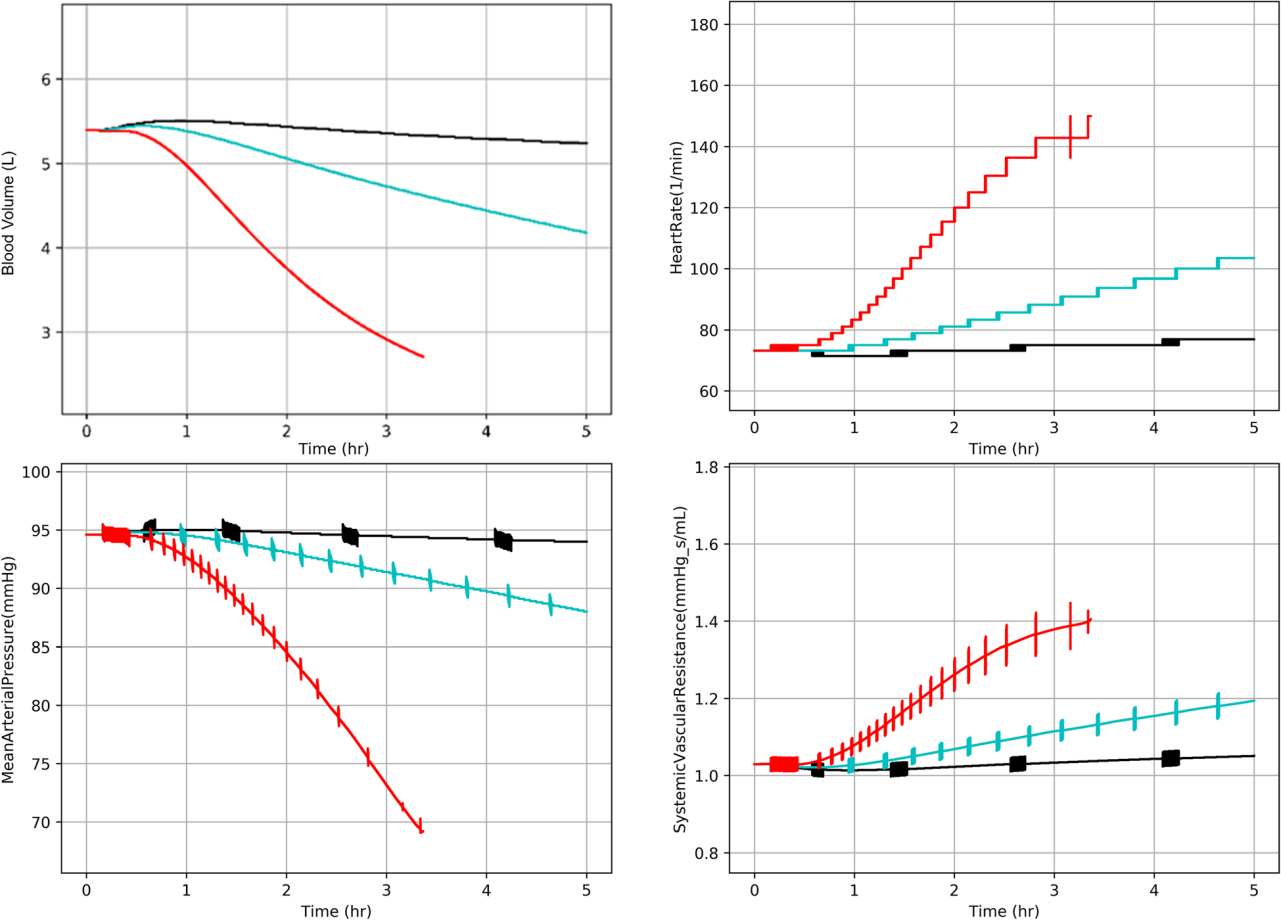
The BioGears response to burns of 10% (black), 25% (blue), and 40% (red) TBSA. Hypotension secondary to volume depletion is opposed by the baroreflex, which increases heart rate and systemic vascular resistance to maintain pressure. This reflex becomes overwhelmed following large burns and leads to irreversible hypovolemia if untreated (red)

BioGears accurately captures the development of interstitial edema and cardiovascular dysfunction as a function of burn severity. A 10% TBSA burn induces minimal fluid loss with no hypotension due to baroreceptor compensation. However, a 25% TBSA burn depletes plasma volume by a liter over 5h, demanding stronger corrective feedback. Both Systemic Vascular Resistance and heart rate increase in this instance to maintain mean arterial pressure and cardiac output. These counteractive measures increase in magnitude following a 40% burn but become overwhelmed, leading to decompensation and loss of half of the vascular volume after 3h [1], Fig. 5.

The burn treatment scenario exhibits reasonable responses to ketamine infusion and fluid challenge. Ketamine depresses the output of the BioGears pain model, causing a reduction in respiratory rate. This drug also induces an increase in blood pressure which, though reasonable in healthy individuals [19], should probably be blunted by the heightened sympathetic state of the burn patient. The initial rate of lactated ringers infusion (250 mL/hr) proves insufficient to reverse fluid loss. However, titrating up the rate by 25% each hour eventually brings urine output (UO) into the target range of 30–50 ml/hour (mL/hr) shortly after the tenth hour of treatment. The restoration of vascular volume leads to improvements in cardiovascular performance as indicated by tachycardia reversal. Cumulatively, the BioGears patient receives 3.4 L of fluid over the first 8h of treatment, which agrees well with the recommendation of literature protocols such as the Parkland formula [1]. However, infusion over the latter hours of treatment becomes too liberal, as the BioGears patient exceeds the recommended twenty-four hour threshold after fifteen simulated hours. This observation likely indicates that the rates of pro-inflammatory cytokines clearance and endothelial healing in the inflammatory model require additional tuning. The BioGears burn model developed under this program has been provided free to the medical training community through the projects GitHub Page (https://github.com/BioGearsEngine/core) with further discussion of the results and model implementation provided in [16]. Future BurnCare modules will focus on BioGears integration for fluid resuscitation and pain management training.

The BurnCare application provides an easy to use, affordable, accurate and readily accessible training platform for burn training that can be used anytime, anywhere, that can supplement current burn care training and lower the barrier to obtain specialized training for health care providers. The free application can be native on an android tablet, allowing for without an internet connection. The training application has the potential to improve the skills of burn non-experienced clinicians in determining the TBSA burned. An accurate TBSA could result in a more accurate determination of severity of burn, more precise burn fluid resuscitation, and determining the correct triage category, appropriate resource allocation such as equipment, supplies and medications, and number of personnel needed to care for patient, potentially improving patient outcomes. Future updates will include modules that cover escharotomy location and depth training, fluid resuscitation, and pain management.

## Conclusions

High incident rates, compounding physiology and unique care create a perfect environment for specialty training. The USAISR Burn Center is the sole treatment facility for burned military casualties within the Department of Defense requiring evacuation over a period of days, with up to 11 different teams providing care. These clinicians have variable levels of knowledge and experience in burn care and many are not acquainted with burn management [20]. The ABLS and other educational courses have been proven to be effective at training for burn specific care but the length of time of the training and the low rate of attendance creates a large care provider population with little to no specialty training. Although initial burn care treatment guidelines are provided to the military medical community through the Joint Trauma System and the ABA ABLS, the BurnCare virtual trainer aims to provide just-in-time baseline training especially for deploying care providers not able to attend ABLS through access of the free tablet application. This project describes Phase I of our development effort. With Phase II funding we plan to add more patient scenarios to practice overall care of a burn patient, more patients to practice TBSA estimations, include information on a resources tab, and incorporate the BioGears physiological engine to improve realism. Upon completion of this project, the BurnCare software will be available on the GooglePlay stores, future updates may target IOS platforms. We also plan to work with collaborators to get the BurnCare game included as a link on several military training web sites such as Deployed Medicine.

## Availability and requirements

**Project Name:** BurnCare Medical Training Application

**Project Homepage:** https://github.com/BioGearsEngine/BurnCare

**Operating Systems:** Android OS, Windows 64bit OS

**Programming Language:** C++

**Other Requirements:** None

**License:** N/A, freely distributed application

**Any restrictions to use by non-academics:** None

## Data Availability

The dataset and application supporting the conclusions of this article (are) available in the BurnCare repository, https://github.com/BioGearsEngine/BurnCare

## Abbreviations

OIF: Operations Iraqi Freedom
OEF: Operation Enduring Freedom
TBSA: Total body surface area
CPG: Clinical practice guideline
USAISR: United States Army Institute of Surgical Research
ABA: American Burn Association
ABLS: American Burn Association
SM: Service Member
SMEs: Subject matter experts
LB: Lund and Browder
IED: Improvised explosive device
TNF: Tumor necrosis factor
DoD: Department of Defense
COP: Colloid osmotic pressure
